# Generation of double Holliday junction DNAs and their dissolution/resolution within a chromatin context

**DOI:** 10.1073/pnas.2123420119

**Published:** 2022-04-22

**Authors:** Han N. Ho, Stephen C. West

**Affiliations:** ^a^DNA Recombination and Repair Laboratory, Francis Crick Institute, London NW1 1AT, United Kingdom

**Keywords:** recombination, repair, BLM, SLX4, GEN1

## Abstract

Four-way DNA intermediates, also known as Holliday junctions (HJs), are formed during homologous recombination and DNA repair, and their resolution is necessary for proper chromosome segregation. To facilitate the biochemical analysis of HJ processing, we developed a method involving DNAzyme self-cleavage to generate 1.8-kb DNA molecules containing either single (sHJ) or double Holliday junctions (dHJs). We show that dHJ DNAs (referred to as HoJo DNAs) are dissolved by the human BLM–TopIIIα–RMI1–RMI2 complex to form two noncrossover products. However, structure-selective endonucleases (human GEN1 and SMX complex) resolve DNA containing single or double HJs to yield a mixture of crossover and noncrossover products. Finally, we demonstrate that chromatin inhibits the resolution of the double HJ by GEN or SMX while allowing BTRR-mediated dissolution.

## Significance

We devised a method involving DNAzyme self-cleavage for the preparation of DNA molecules containing double Holliday junctions (dHJs) that are separated by 746 bp. The DNAs can be prepared in large amounts suitable for in vitro analysis of enzymes required for the processing of recombination intermediates. We show that the dHJ DNA is dissolved efficiently by the BLM–TopIIIα–RMI1–RMI2 (BTRR) complex to produce noncrossover products, whereas nucleolytic resolution by GEN or SMX results in both crossover and noncrossover products. Following nucleosome assembly, the chromatinized dHJ structures are preferentially dissolved by BTRR. These new Holliday junction substrates will provide a valuable resource for the mechanistic analyses of the way in which recombination intermediates are processed.

Homologous recombination (HR) provides an important mechanism for the repair of DNA double-stranded breaks and the restoration of broken replication forks (1). Recombination in mitotic cells generally occurs between sister chromatids and can lead to the formation of DNA intermediates in which the sisters are covalently linked by four-way DNA junctions, known as Holliday junctions (HJs) (2). Failure to process these DNA intermediates leads to improper chromosome segregation and cell death (3, 4). Recombination also plays an important role in meiotic cells, when interactions occur between homologous chromosomes, and is responsible for the generation of genetic diversity.

In mitotic cells, HJs are primarily processed by “dissolution,” in which two adjacent HJs (double Holliday junctions [dHJs]) converge in an adenosine triphosphate (ATP)-hydrolysis–dependent reaction to form a hemicatenane that is subsequently decatenated by topoisomerase action. In human cells, this two-step process involves the BLM–topoisomerase IIIα–RMI1–RMI2 (BTRR) complex (5–7). In yeast, similar reactions are driven by the Sgs1–Top3–Rmi1 (STR) complex (8, 9). Dissolution yields exclusively noncrossover products, which help to maintain the heterozygous state, as a loss of heterozygosity can cause cancer development (10). Mutations in the *BLM* gene are linked to a human inherited disorder known as Bloom syndrome, which is characterized by short stature, sensitivity to sunlight, and a greatly increased risk of a broad range of cancers (11, 12). In the clinic, patients with Bloom syndrome are diagnosed by a cytogenetic test that detects elevated levels of sister chromatid exchanges. Pathogenic mutations in the *TOP3A* and *RMI1* genes also cause a Bloom syndrome–like disorder, consistent with the fact that these genes participate in the same molecular pathway (12).

Persistent HJs that escape processing by BTRR, and single Holliday junctions (sHJs), are resolved by structure-selective endonucleases (SSEs), which specifically recognize and cleave HJs by mediating a nucleolytic attack on two opposing strands at the junction point (2). In humans, these endonucleases include GEN1 and the SMX trinuclease, comprising SLX1–SLX4–MUS81–EME1–XPF–ERCC1 (3, 13–15). Unlike dissolution, resolution gives rise to both crossover and noncrossover products, thereby elevating the frequency of sister chromatid exchanges and increasing the potential for loss of heterozygosity.

In contrast to BTRR-mediated dHJ dissolution, which is active throughout the cell cycle, the actions of SMX and GEN1 are tightly regulated. Firstly, SMX complex formation is restricted to prometaphase, as it is dependent upon the phosphorylation of EME1 by CDK1 and PLK1, which stimulates its association with the SLX4 scaffold (15, 16). Secondly, GEN1 is mainly sequestered from the cell nucleus and gains access to DNA after the breakdown of the nuclear envelope during cell division (17).

Despite the importance of the BTRR complex in maintaining genetic stability, a detailed picture of dissolution is lacking. Mechanistic studies, using protein complexes from various organisms, led to a model in which two HJs are converged by the branch migration activity of the BLM helicase (18–20). Convergent migration generates positive supercoiling that is relaxed by topoisomerase IIIα and generates a hemicatenane that is processed by topoisomerase IIIα with the aid of RMI1–RMI2 (6–9, 21).

Studies of dHJ dissolution have utilized two model systems: 1) a small dHJ prepared by annealing two synthetic oligos (5–7), and 2) a larger plasmid-sized molecule in which two HJs are separated by 165 bp (20, 22). However, the small size of the synthetic DNA substrate eliminates any possibility for branch migration as the two HJs are separated by only 14 bp, raising concerns as to whether these substrates recapitulate the physiological aspects of dissolution (19). The plasmid-sized substrate has been utilized for the dissolution of dHJs by *Saccharomyces cerevisiae* STR, *Drosophila melanogaster* BTR, and more recently human BTRR complex (8, 20, 23). However, there is the significant drawback that generation of this substrate is laborious (taking several weeks), requires purified Cre recombinase and reverse gyrase, and leads to low yields of product.

To facilitate mechanistic analysis of dissolution and resolution, we developed a rapid and scalable methodology to prepare a 1.8-kb DNA containing single or double HJs. In the dHJ molecules, the two HJs are separated by a maximum of 746 bp of homologous sequence, allowing the two HJs to migrate within the region of homology. We demonstrate that these dHJ molecules are efficiently dissolved by the human BTRR complex to generate noncrossover products. We also show that GEN1 or SMX resolves the single or double HJs to yield the expected mixture of crossover and noncrossover products. Finally, we find that GEN1/SMX are unable to resolve HJs on chromatinized templates, whereas BTRR-mediated dissolution events are unaffected by nucleosome assembly, potentially indicative of an additional level of regulatory control that favors dissolution over resolution.

## Results and Discussion

### Design of HR Intermediates Containing Double Holliday Junctions.

**Fig. 1.**
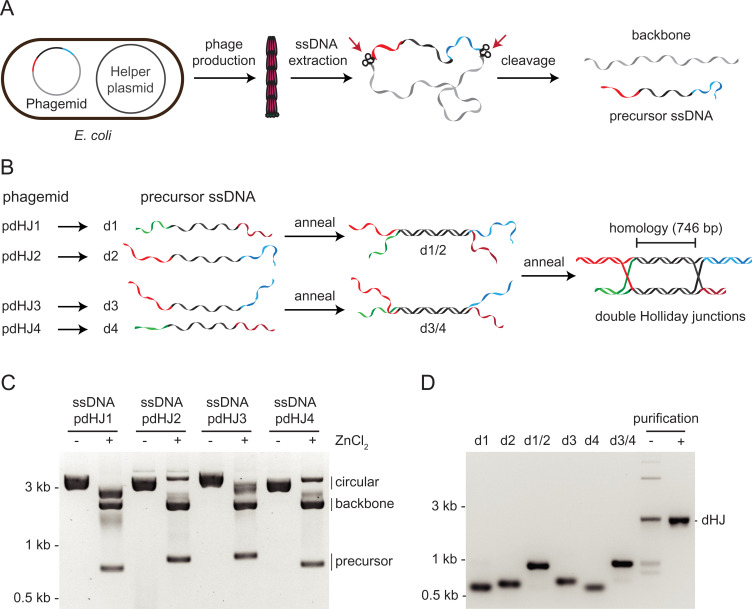
Generation of DNA molecules containing dHJs. (*A*) Schematic diagram indicating ssDNA production methodology. Circular ssDNA is produced by *E. coli* cells carrying phagemids pdHJ1, pdHJ2, pdHJ3, or pdHJ4, and a helper plasmid expressing M13 viral proteins. The circular ssDNAs isolated from each phage contain two DNAzyme self-cleavage cassettes, which cleave their own phosphodiester bonds in the presence of Zn^2+^. This enables the separation of precursor ssDNA (colored) from phagemid backbone ssDNA (gray). (*B*) Schematic showing the generation of dHJs by annealing four precursor ssDNAs (d1–d4). The precursor ssDNAs (d1–d4) are generated from their corresponding phagemids (pdHJ1–pdHJ4). Following annealing and purification, the two HJs are separated by a 746-bp region of homology (indicated in black). (*C*) Gel electrophoretic analysis showing the cleavage of circular ssDNA in the presence of ZnCl_2_ to generate linear precursor ssDNAs d1–d4. (*D*) Gel electrophoretic analysis of purified precursor ssDNAs (d1–d4) and their annealed products: d1/2, d3/4, and dHJ molecules. The dHJ DNA was purified by gel electrophoresis followed by electroelution.

We set out to generate a dHJ substrate that could be rapidly prepared in significant quantities by annealing four single-stranded DNAs (ssDNAs) (Fig. 1 *A* and *B*). To recapitulate physiological aspects of dissolution in which two Holliday junctions are separated by hundreds to thousands of nucleotides (nt), we designed the precursor ssDNAs to be between 800 and 1,000 nt in length. The precursor DNAs were cloned into a phagemid backbone containing the *f1* origin of replication. This allowed the phagemid to be packaged in the form of circular ssDNA by *Escherichia coli* cells expressing M13 viral proteins from a helper plasmid (Fig. 1*A*). Following ssDNA extraction, the precursor DNAs were excised from the single-stranded circles using Zn^2+^-dependent DNAzyme self-cleavage cassettes (Fig. 1 *A* and *C* and *SI Appendix*, Fig. S1*A*) (24). The precursor ssDNAs were then purified from the backbone ssDNA, such that the four precursor ssDNAs (d1–d4) could be combined by thermal annealing (Fig. 1*B*). First, two pairs of partially complementary ssDNAs were annealed in separate reactions: d1 to d2 and d3 to d4. Each reaction created a 746-bp duplex DNA intermediate (d1/2 or d3/4) with single-stranded tails. The duplex regions in d1/2 and d3/4 (shown in black, Fig. 1*B*) are homologous to each other. In the second annealing step, d1/2 and d3/4 were added together so that the complementary sequences of the single-stranded tails annealed to form duplex DNA. The resultant DNAs contained dHJs separated by a maximum of 746 bp and were purified by agarose gel electrophoresis and electroelution (Fig. 1 *B* and *D*).

### Dissolution of dHJs by the BTRR Complex.

**Fig. 2.**
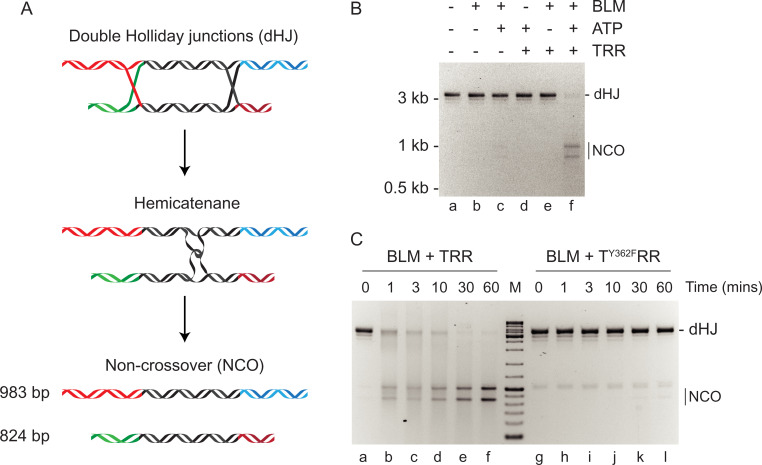
Dissolution of double Holliday junctions by human BTRR complex. (*A*) Schematic diagram showing the dissolution of dHJ DNA to yield noncrossover products. ATP-dependent branch migration mediated by BLM helicase gives rise to a hemicatenane that is processed by topoisomerase IIIα–RMI1–RMI2. (*B*) Dissolution of dHJ DNA by the human BTRR complex and dependence on nucleotide cofactor. Reactions contained dHJ DNA (3 nM), BLM (100 nM), TRR (300 nM), and ATP (5 mM) where indicated. Products were analyzed by agarose gel electrophoresis and visualized by ethidium bromide staining. (*C*) Time course showing the dissolution of dHJs (3 nM) by BTRR complex (100 nM BLM and 300 nM wild-type or mutant TRR). The mutant T^Y362F^RR complex failed to catalyze dissolution. M, DNA size markers. Products were analyzed as in *B*.

To validate the dHJ molecules, we first analyzed them in dissolution assays using recombinant human BLM helicase and the topoisomerase IIIα–RMI1–RMI2 (TRR) complex. For these experiments, BLM helicase and the TRR complex were independently expressed and purified from baculovirus-infected insect cells (*SI Appendix*, Fig. S2). A characteristic of dissolution is the generation of two noncrossover products (5); based on the design of the dHJ molecules, we expected the noncrossover products to be 983 and 824 bp in length (Fig. 2*A*). We observed the formation of two products in the presence of BLM helicase, ATP, and the TRR complex (Fig. 2*B*, lane *f*). Given these products are consistent with the expected sizes of noncrossover products and that their formation requires the BLM helicase, ATP, and the TRR complex, we conclude that the dHJs serve as a substrate for dissolution by the BTRR complex. A time course of dissolution by BTRR is shown in Fig. 2*C*, lanes *a*–*f*. We did not observe dissolution when the dHJs were incubated with BLM in the presence of a mutant T^Y362F^RR complex, in which the catalytic residue Y362 (5, 25) in topoisomerase IIIα was replaced with phenylalanine (Fig. 2*C*, lanes *g*–*l*).

### Resolution of the dHJs by GEN1 and the SMX Complex.

**Fig. 3.**
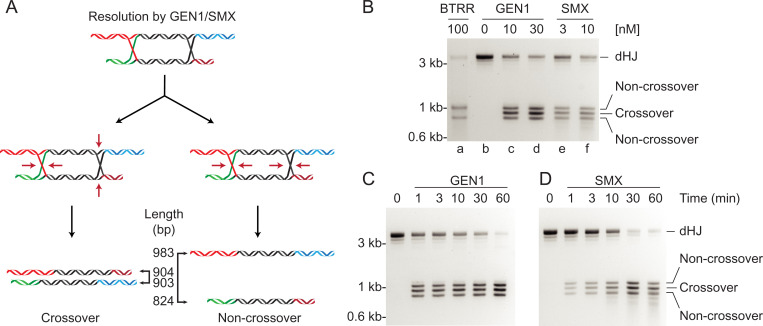
Resolution of double Holliday junctions by GEN1 and the SMX complex. (*A*) Schematic diagram indicating the formation of both crossover and noncrossover products dependent on the orientation of resolution (red arrows). The sizes of each product are indicated. (*B*–*D*) Resolution of dHJ DNA by GEN1 and SMX yields a mixture of crossover and noncrossover products, whereas BTRR (100 nM BLM helicase and 300 nM TRR complex) produces only noncrossover products. Reactions contained dHJ (3 nM) and the indicated concentrations of GEN1 or SMX (*B*), or dHJ (6 nM) (*C* and *D*), and GEN1 (50 nM) (*C*), or SMX (10 nM) (*D*). Reactions were incubated at 37 °C for 20 min (*B*) or the indicated times (*C* and *D*) and products analyzed by agarose gel electrophoresis.

Next, we characterized the resolution of dHJ molecules using recombinant human GEN1 and SMX. As nucleolytic cleavage can occur in two different orientations (Fig. 3*A*), we expected GEN1 and SMX to cleave the dHJs to yield a mixture of crossover and noncrossover products (26). Based on the design of the dHJ molecules, we anticipated the lengths of the crossover products to be 903 and 904 bp, respectively, whereas the noncrossover product would be 824 and 983 bp. Consistent with this, we observed three product bands following cleavage; the top and the bottom bands correspond to the noncrossover products, whereas the middle band represents the two crossover products (Fig. 3*B*, lanes *c*–*f*). Time-course experiments showed that both GEN1 and SMX efficiently cleaved the dHJ DNA, as the mixture of crossover and noncrossover products increased as a function of time (Fig. 3 *C* and *D*).

### Single HJ Molecules Are Resolved by GEN1 and the SMX Complex.

**Fig. 4.**
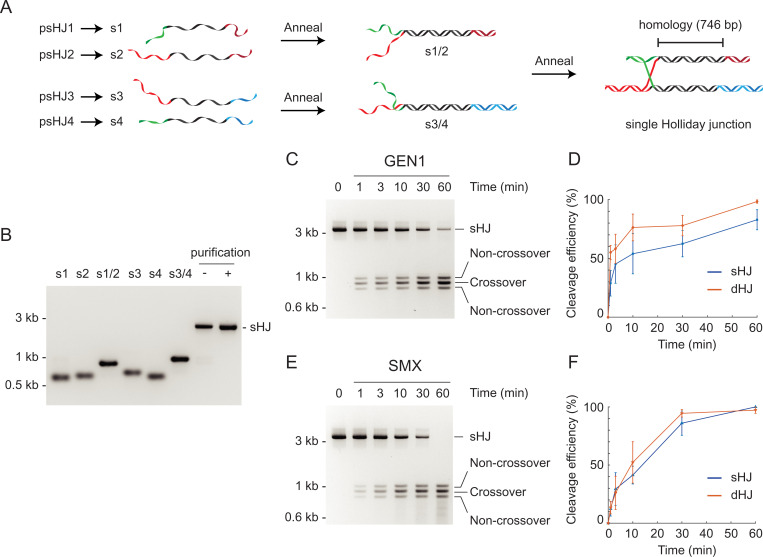
Resolution of sHJs by GEN1 and SMX. (*A*) Schematic diagram showing the generation of sHJs by annealing four linear ssDNAs (s1–s4). The 746-bp homologous region (shown in black), through which the HJ can migrate, is the same as that present in the dHJ DNA. (*B*) Precursor ssDNAs s1–s4, made by a procedure similar to that shown in Fig. 1*A*, and their annealed products. (*C*) Resolution of sHJ (6 nM) by GEN1 (50 nM), as determined by agarose gel electrophoresis. (*D*) The efficiency of sHJ cleavage by GEN1, shown in *C*, was quantified and compared with dHJ cleavage (Fig. 3*C*). (*E* and *F*) As in *C* and *D*, except using SMX complex (10 nM). The efficiency of dHJ cleavage was quantified from Fig. 3*D*. Error bars, SEM (*n* = 3).

In contrast to the dHJs, sHJs can only be processed by the resolution pathway and not by dissolution. To facilitate the biochemical and biophysical analysis of sHJ resolution, we modified the sequence of the precursor ssDNAs to generate DNA molecules containing a single HJ (*SI Appendix*, Fig. S1*C*). The precursor ssDNAs (s1–s4) were annealed in two steps and the sHJ product was purified by agarose gel electrophoresis followed by electroelution (Fig. 4 *A* and *B*). We found that the sHJ molecules were efficiently cleaved by GEN1 (Fig. 4 *C* and *D*) and SMX (Fig. 4 *E* and *F*) to yield a mixture of crossover products (903 and 904 bp) and noncrossover products (824 and 983 bp). Comparisons of the rates of cleavage of equimolar concentrations of dHJ and sHJ molecules by GEN1 and SMX are shown (Fig. 4*D* and *F*). No significant differences were observed in cleavage efficiency.

### Chromatin Inhibits Resolution but Not Dissolution of the dHJs.

**Fig. 5.**
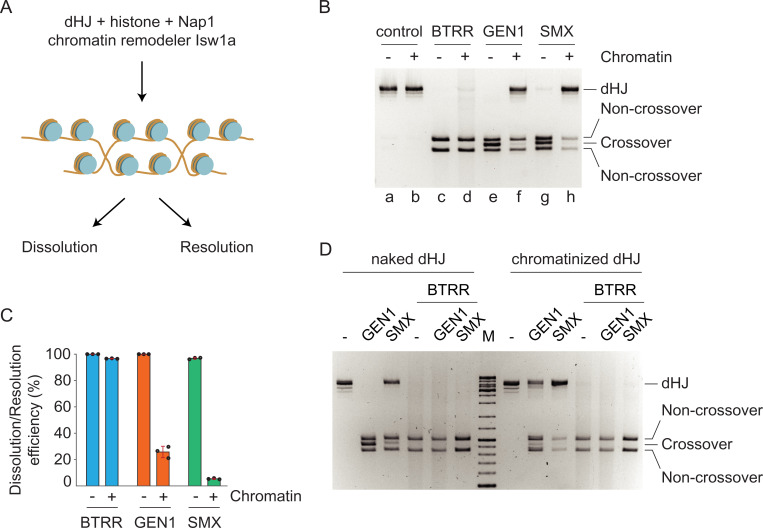
Dissolution and resolution of dHJ DNA within a chromatin context. (*A*) Schematic diagram showing the experimental design. Regularly spaced chromatin was prepared by depositing histones on dHJ DNA using the histone chaperone Nap1 and the chromatin remodeler Isw1a. (*B*) HJ resolution by GEN1 and SMX is inhibited by the presence of chromatin, whereas BTRR-mediated dissolution is unaffected. Reactions contained naked or chromatinized dHJ DNA (2.6 nM) with BLM helicase (124 nM) and TRR (320 nM), or GEN1 (40 nM), or SMX (40 nM). Reactions were incubated for 1 h at 37 °C and the products analyzed by agarose gel electrophoresis. (*C*) Quantification of dissolution and resolution efficiency in *B*. Black circles represent individual repeats. Error bars, SD (*n* = 3). (*D*) Competition between resolution and dissolution. GEN1 (30 nM), SMX (30 nM), BTRR (124 nM BLM and 320 nM TRR), or premixed enzymes, were added to naked or chromatinized dHJ DNA (1.3 nM). Reactions were incubated for 1 h at 37 °C and the products analyzed by agarose gel electrophoresis.

Chromatin remodeling is a prerequisite for efficient HR in cells, but how chromatin impacts HJ resolution and dissolution remains unknown. To understand whether resolution and dissolution can occur on chromatinized HJs, we assembled histones onto dHJ molecules with the aid of the histone chaperone Nap1 and the chromatin remodeler Isw1a (Fig. 5*A*) (27, 28). Partial digestion with micrococcal nuclease demonstrated the regular spacing of histone octamers on dHJ molecules (*SI Appendix*, Fig. S3). We found that BTRR dissolved the dHJs present on the chromatinized DNA (97 ± 1% compared to naked templates) to produce the expected noncrossover products (Fig. 5*B*, lanes *c* and *d*, Fig. 5*C*, and *SI Appendix*, Fig. S4). A comparison of dHJ cleavage, by various concentrations of BTRR, in the presence and absence of chromatin is shown in *SI Appendix*, Fig. S4. In contrast, the presence of chromatin inhibited the resolution of the dHJs by GEN1 (Fig. 5*B*, lanes *e* and *f*) and SMX (Fig. 5*B*, lanes *g* and *h*). Quantifications revealed that the resolution efficiencies on chromatinized dHJs, in comparison with naked DNA, were only 26 ± 4% for GEN1 and 5.6 ± 0.5% for SMX (Fig. 5*C*). Curiously, of the fraction of chromatinized dHJs that underwent resolution, the majority of products were noncrossovers (Fig. 5*B*, lanes *f* and *h*). The reason for this preference is currently unknown. Finally, when a mixture of BTRR and GEN1, or BTRR and SMX, were incubated together in the presence of naked or chromatinized dHJs, we observed only noncrossover products (Fig. 5*D*). These results show that HJ dissolution predominates over resolution with regard to dHJ processing.

In summary, we have developed a method to generate 1.8-kb DNA molecules containing double or single HJs. The 746-bp region of homology in the dHJ substrate allows the junctions to undergo convergent branch migration during dissolution. Hence, these molecules can serve as an excellent model substrate to facilitate studies into the mechanism of dissolution by BTRR. However, since RPA stimulates the unwinding of partially single-stranded templates by BLM (29, 30), we wish to point out that these substrates are unlikely to be useful in studies containing RPA, as the dHJs would be unwound from their ssDNA tails. To overcome this potential issue, it would be relatively straightforward to fill in the ssDNA tails or redesign the initial annealing templates to make the dHJs completely double stranded.

We also showed that dHJ molecules were dissolved by the human BTRR complex to yield noncrossover products, whereas dHJ and sHJ molecules were resolved by GEN1 or SMX to form a mixture of noncrossover and crossover products. We also found that chromatin inhibits the resolution of dHJs, whereas BTRR-mediated dissolution is less unperturbed. These observations indicate that chromatin presents a physical block that denies GEN1 and SMX access to HJs and implies that resolution in vivo is likely to require chromatin remodeling activities. Surprisingly, the products that did form by resolution of the chromatinized templates were restricted to noncrossovers. The reason for this apparent bias in cleavage is presently unknown and requires further investigation. In contrast to the resolution reactions, we found little inhibition of dissolution, indicating that BLM helicase can itself remodel chromatin during HJ branch migration to allow dissolution of the chromatinized dHJs. In cells, dissolution is the primary pathway to process HJ intermediates, whereas SMX and GEN1 are regulated temporally and spatially. Our observations suggest that chromatin may present an additional layer of regulation that limits the activity of the endonucleases and favors dissolution over resolution.

## Materials and Methods

### Plasmids for ssDNA Production.

All phagemids contain the *f1* origin of replication that allows their packaging into M13 viral particles in the form of ssDNA (*SI Appendix*, Table S1). The assembly of double HJs requires four of these phagemids: pdHJ1, pdHJ2, pdHJ3, and pdHJ4. Similarly, the single HJs were prepared from psHJ1 (same as pdHJ1), psHJ2, psHJ3 (same as pdHJ3), and psHJ4. The phagemid sequences are shown in *SI Appendix*, Tables S2–S7.

Phagemids were created by assembling synthetic DNA fragments (IDT) onto a phagemid backbone [pBluescript II SK(+), Agilent], using the Gibson assembly (NEB). Specifically, pBluescript II SK(+) was digested with *Sap*I and *Pvu*II (NEB). Synthetic DNA fragments were designed with five elements (*SI Appendix*, Fig. S1*A*): 1) 25- to 30-bp homology to pBluescript II SK(+) backbone (*Sap*I site); 2) the first DNAzyme self-cleavage cassette; 3) precursor DNA; 4) the second DNAzyme self-cleavage cassette; and 5) 25- to 30-bp homology to pBluescript II SK(+) backbone (*Pvu*II site). Homologous sequences 1 and 5 allow the precursor DNA to be inserted with the intended orientation into the backbone during Gibson assembly reactions (31) (note that the original *Sap*I site near *ori* was lost in the resultant phagemids). The sequence of the precursor DNA in pdHJ1, d1, was generated using a random sequence generator (GC content of 50%). The sequences of the other precursor DNAs were designed based on d1, so that they can anneal to generate DNA molecules containing Holliday junctions.

The M13 helper plasmid (HP4_M13) was a gift from Hendrik Dietz, Technical University of Munich, Munich, Germany (Addgene plasmid 120340).

### Single-Stranded DNA Produced in *E. coli* Using the M13 Helper Plasmid.

Single-stranded DNA was produced in *E. coli* DH5α as described (24). DH5α cells harboring a phagemid and the helper plasmid HP4_M13 were grown at 37 °C with vigorous shaking in 2× YT supplemented with 5 mM MgCl_2_, 30 μg/mL kanamycin, and 50 μg/mL ampicillin. Starter cultures (8 mL) were inoculated with a few freshly transformed colonies for 8 h. These cultures were used to seed 800-mL cultures grown in 5-L flasks for 14 to 16 h, during which viral particles containing ssDNA were extruded into the media.

The media were cleared of bacteria by centrifugation twice (6,000 × *g* for 15 min at 4 °C). The viral particles were precipitated following the addition of PEG-6000 (Sigma-Aldrich) and NaCl (Sigma-Aldrich), each to 40 g per liter of supernatant. The mixture was incubated for 1 h at 4 °C. The phage particles were harvested by centrifugation (8,000 × *g* for 20 min at 4 °C) and resuspended in 6 mL of TE buffer (50 mM Tris-HCl pH 8.0, 1 mM EDTA). The ssDNA was extracted using alkaline lysis. Briefly, one volume of lysis buffer (0.2 M NaOH/1% Sodium dodecyl sulfate) was added and mixed with the viral particle suspension by inversion. The mixture quickly turned clear. Immediately, 1.5 volumes of neutralizing buffer (3 M potassium acetate, pH 5.5) were added and mixed by inversion. Protein precipitates were removed by centrifuging twice (15,000 × *g* for 15 min at 18 °C). To extract DNA from the supernatant, isopropanol was added (0.7 volume of the supernatant), followed by centrifugation (15,000 × *g* for 30 min at 18 °C) in 50-mL Falcon tubes. The ssDNA precipitate was washed with 40 mL of ice-cold 70% ethanol (15,000 × *g* for 10 min at 18 °C) to remove residual salt. The ethanol was then discarded, and ssDNA was resuspended in 5 mL of TE buffer (pH 8.0). A yield of 6 to 10 mg of ssDNA per liter of culture is expected.

### Cleavage of Circular ssDNA and Purification of Precursor ssDNA.

Cleavage reactions were performed as previously described (24). Large-scale cleavage reactions (10 mL) contained circular ssDNA in (0.5 to 1 μM; 5 to 10 mg) in 50 mM Hepes pH 7.0, 100 mM NaCl, and 4 mM ZnCl_2_. Incubation was at 37 °C, either overnight or for 24 h. Following cleavage, DNA was precipitated with ethanol and resuspended in 5 mL of TE, pH 8.0.

Following cleavage, the precursor ssDNA (d1–d4, s1–s4) was separated from backbone ssDNA and other DNA contaminants by gel electrophoresis and electroelution as described (32). Typically, a 180-mL 2% (wt/vol) agarose/Tris-acetate-EDTA (TAE) gel was cast in a 15 × 10 cm gel tray (Bio-Rad 1704416) with a single large sample well. Following the addition of DNA loading dye, about 1.5 mg of cleaved products was loaded and gel electrophoresis was carried out in TAE at 5 V/cm for 2 h at 4 °C. A section of the gel was cut out and stained with ethidium bromide (Sigma-Aldrich) to determine the part of the gel for excision. In these conditions, precursor and backbone ssDNAs migrated at similar rates to dsDNA of 600 to 800 bp and 1.8 kb, respectively. The 1-cm gel fragments contained the precursors were excised and placed in SnakeSkin dialysis tubes (Thermo Fisher, 10 K molecular-weight cutoff, 16-mm diameter) containing 7 mL of TAE. Next, the tube was submerged in TAE buffer in a gel electrophoresis tank, and DNA was eluted from the gel for 1 h at 5 V/cm at 4 °C. DNA was then precipitated with ethanol and the pellets were resuspended in 500 μL of TE buffer (pH 8.0). Typically, 200 to 300 μg of each precursor DNA was obtained.

### Generation of DNA Molecules Containing Single and Double Holliday Junctions.

DNA molecules containing dHJs were generated from precursor ssDNAs (d1, d2, d3, and d4) by thermal annealing. Precursor DNA d1 (831 nt), d2 (990 nt), d3 (990 nt), and d4 (831 nt) (*SI Appendix*, Fig. S1*B*) were obtained from pdHJ1, pdHJ2, pdHJ3, and pdHJ4, respectively. In separate tubes, 25 to 30 nM of d1 and d2 were annealed to form d1/2, while d3 and d4 were annealed to form d3/4. Annealing reactions were carried out in 30 μL or 12 mL of annealing buffer (10 mM Tris⋅HCl pH 7.5, 10 mM MgCl_2_, 50 mM NaCl). For each new batch of ssDNA, pilot reactions (30 μL) were performed, where the molar ratio of d2/d1 (and d4/d3) varied (0.8:1, 0.9:1, 1:1, 1.1:1, and 1.2:1), to establish the optimal conditions for large-scale reactions (12 mL). Using a heat block, the reactions were heated at 90 °C for 5 min before the heating device was switched off and the reactions were cooled slowly to ambient temperature (4 h or overnight). The annealed DNAs (d1/2 and d3/4) were precipitated with ethanol and resuspended in 500 μL of TE buffer (pH 8.0). Next, d1/2 and d3/4 were purified from unreacted ssDNA by 2% agarose gel electrophoresis followed by electroelution as described above. Finally, d1/2 and d3/4 (6 nM each) were annealed to generate dHJs in a reaction containing annealing buffer. The concentrations of d1/2 and d3/4 were kept low to minimize the formation of multimer DNA species. Pilot reactions (200 μL) and large-scale reactions (5× 9-mL reactions in 15-mL Falcon tubes) were heated at 75 °C for 5 min before the heating device was switched off to slowly cool down to ambient temperature. The annealed DNAs were precipitated with ethanol and resuspended in 500 μL of TE buffer (pH 8.0). Finally, dHJ molecules were purified from other DNA species by 2% agarose gel electrophoresis, followed by electroelution. Gel electrophoresis was performed for 4 h at 4 V/cm at 4 °C and the TAE buffer was replaced every 90 min to avoid overheating. During electroelution, dHJs were eluted for 2 h at 4 V/cm at 4 °C. Finally, dHJ DNA was precipitated with ethanol and resuspended in TE buffer (pH 8.0). Typically, 100 μg of dHJ DNA was obtained.

DNA molecules containing sHJs were generated using a similar method, by thermally annealing precursor ssDNAs (s1–s4). Precursor DNA s1 (831 nt), s2 (911 nt), s3 (990 nt), and s4 (910 nt) (*SI Appendix*, Fig. S1*C*) were obtained from psHJ1, psHJ2, psHJ3, and psHJ4, respectively. Briefly, s1 and s2 were annealed to form s1/2, while s3 and s4 were annealed to form s3/4. Reactions contained 25 to 30 nM of each precursor ssDNA in 12 mL of annealing buffer. Finally, s1/2 and s3/4 were annealed to generate sHJ DNA (8 nM of s1/2 and s3/4 in 18-mL reactions), followed by gel electrophoresis and electroelution. Typically, more than 100 μg of sHJ DNA was obtained.

### Plasmids and Protein Purification.

pFL-TRR (pFL-_MBP_RMI1-RMI2-_His_TOP3A) was created in a series of steps. First, the *RMI1* sequence was amplified from pDEST15-_GST_RMI1 (33) and cloned into pFL (34) using the *Xho*I and *Sph*I sites, and then the *RMI2* gene was inserted at the *Bss*HII site to create pFL-_GST_RMI1-RMI2. Next, the GST tag was replaced with the MBP tag to generate pFL-_MBP_RMI1-RMI2.

In parallel, the *TOP3A* and *BLM* genes were cloned into pUCDM (34) at the *Rsr*II and *Xba*I sites, and *Xho*I and *Sph*I sites, respectively, to generate pUCDM-_His_BLM-TOP3A. The *BLM* gene was eventually removed, while a His tag was introduced directly upstream of *TOP3A* using the Gibson assembly (*Pme*I and *Acc*I sites) to generate pUCDM-_His_TOP3A. Finally, pFL-TRR was created from pFL-_MBP_RMI1-RMI2 and pUCDM-_His_TOP3A using Cre-mediated recombination (NEB). Similarly, the mutant gene encoding TopoIIIα^Y362F^ was cloned into pUCDM to generate pUCDM-TOP3A^Y362F^, which was then recombined into pFL-_MBP_RMI1-RMI2 to generate pFL-T^Y362F^RR. pFL-TRR and pFL-T^Y362F^RR were used to generate bacmids and P1 and P2 baculovirus for expression of the wild-type and mutant TRR complexes in *Sf*9 cells.

Wild-type TRR complex was purified from ∼2.5 × 10^9^ *Sf*9 cells infected with pFL-TRR P2 baculovirus at an multiplicity of infection of 1 for 66 h at 28 °C (150 rpm). Cell pellets were resuspended in three pellet volumes of hypotonic lysis buffer (50 mM Tris⋅HCl pH 7.5, 1 mM EDTA, 5 mM β-mercaptoethanol and 1× Halt protease and phosphatase inhibitor mixture; Thermo Fisher) with stirring at 4 °C for 30 min. Glycerol was added to a final concentration of 16.7%, then NaCl (5 M) was added to reach a final concentration of 300 mM. The lysate was clarified using centrifugation (15,000 × *g*, 30 min at 4 °C) and the supernatant was transferred to a 100-mL bottle containing 10 mL of amylose resin (NEB) that had been equilibrated with wash buffer (10% glycerol, 50 mM Tris⋅HCl pH 7.5, 5 mM β-mercaptoethanol, and 0.3 M NaCl). The mixture was incubated on a roller for 1 h at 4 °C and then transferred to 50-mL Falcon tubes. The beads were washed four times with 40 mL of wash buffer containing 1 M NaCl, before being transferred to two 20-mL columns (Bio-Rad 7321010) and washed for 1 h in wash buffer containing 1 M NaCl then four column volumes (CVs) of wash buffer containing 0.55 M NaCl and 4 CVs of wash buffer containing 0.1 M NaCl. The TRR complex was eluted with three CVs of elution buffer (wash buffer containing 0.1 M NaCl supplemented with 20 mM maltose) with a 5-min incubation between each elution. Eluates were pooled and the MBP tag was cleaved at 4 °C for 4 h using 3C protease (added to 40 μg per 100 μg of TRR mix). The mixture was added to 1 mL of heparin Sepharose fast flow beads (Cytiva) that had been equilibrated with elution buffer. The mixture was transferred to a 10-mL Poly-Prep column (Bio-Rad) and the beads were washed for 1 h with wash buffer containing 0.1 M NaCl. The TRR complex was eluted with wash buffer containing 0.3 M NaCl. Eluates were pooled and 20-μL aliquots were snapped frozen in liquid nitrogen and stored in −80 °C.

The mutant TRR complex, comprising topoisomerase IIIα (Y362F) and wild-type RMI1–RMI2 (T^Y362F^RR), was expressed in *Sf*9 cells and purified using amylose and heparin resins as above.

BLM helicase was expressed in *Sf*9 cells (using baculovirus derived from plasmid pFB-_MBP_BLM_His_) and purified as described (35). GEN1 was purified as described previously (36), except protein expression was induced by the addition of D-(+)-galactose (to 2%) to yeast cultures in early exponential phase (OD_600_ ∼0.4). SMX (_V5_SLX1-_STREP_SLX4-MUS81-_FLAG_EME1-_HIS6_XPF-ERCC1) was purified as described (37). Yeast histone octamers, Nap1 and Isw1a, were purified as described (28, 38).

### Holliday Junction Dissolution.

Dissolution assays were carried out in buffer containing 20 mM Tris⋅HCl pH 7.5, 1 mM dithiothreitol (DTT), 200 ng/μL of bovine serum albumin (BSA), 2 mM MgCl_2_, and 5 mM ATP. The reaction contained dHJ DNA (3 nM), and where indicated, BLM helicase (100 nM) and TRR or T^Y362F^RR complex (300 nM). Reactions were incubated at 37 °C for 10 min, or for the indicated times, before being stopped with the addition of 5× stop buffer (4% SDS, 100 mM Tris⋅HCl, pH 7.5, and 10 mg/mL proteinase K) followed by a 15-min incubation at 37 °C.

### Holliday Junction Resolution.

Resolution assays were carried out in resolution buffer containing 50 mM Tris⋅HCl, pH 8.0, 1 mM MgCl_2_, 1 mM DTT, and 100 ng/μL BSA. Unless indicated otherwise, reactions contained sHJ or dHJ and the indicated concentrations of GEN1 or SMX. Reactions were incubated at 37 °C for 20 min or the indicated times before being stopped with the addition of 5× stop buffer (4% SDS, 100 mM Tris⋅HCl, pH 7.5, and 10 mg/mL proteinase K) followed by a 15-min incubation at 37 °C.

### Dissolution and Resolution of Chromatinized dHJ.

Chromatin assembly was performed as described (27, 28). In 1.5-mL tubes, 40-μL reactions containing Nap1 (3.66 μM), yeast histone octamer (0.38 μM), and Isw1a (3 nM) were incubated on ice for 30 min in 5/50 buffer (10 mM Hepes-KOH pH 7.6, 5 mM MgCl_2_, 50 mM KCl, 0.5 mM EDTA, 10% glycerol, and 100 ng/μL BSA). An ATP regeneration system (creatine kinase to 140 ng/μL, phosphocreatine to 45 mM, and ATP to 3 mM) and dHJ DNA (to 11 nM) were added and the mixture was incubated at 30 °C for 4 h. The efficiency of chromatin assembly was assayed by digesting chromatinized dHJ (220 ng) with 300 units of micrococcal nuclease (2,000 units/μL, NEB) for 5 or 15 min at 37 °C in micrococcal nuclease reaction buffer (50 mM Tris⋅HCl pH 7.9, and 5 mM CaCl_2_), and the digested products were analyzed by 1.3% agarose gel electrophoresis (*SI Appendix*, Fig. S2). Control reactions involving naked dHJs were set up in the same manner except histone was replaced with histone buffer (20 mM Tris⋅HCl pH 8.0, 2 M NaCl, 0.1 mM EDTA, and 10 mM β-mercaptoethanol).

Following chromatin assembly, dissolution or resolution reactions were set up with the respective dissolution or resolution buffer and contained naked or chromatinized dHJ (2.6 nM), BLM helicase (124 nM), and TRR (320 nM), or GEN1 (40 nM) or SMX (40 nM). Dissolution/resolution competition reactions contained naked or chromatinized dHJ (1.3 nM), and where indicated, BLM (124 nM), TRR complex (320 nM), GEN1 (30 nM), or SMX (30 nM) in combined dissolution/resolution buffer (20 mM Tris⋅HCl pH 7.5, 1 mM DTT, 200 ng/μL of BSA, 1 mM MgCl_2_, and 1 mM ATP). Reactions were incubated at 37 °C for 1 h before being stopped with the addition of 5× stop buffer (4% SDS, 100 mM Tris⋅HCl, pH 7.5, and 10 mg/mL proteinase K) followed by a 45-min incubation at 50 °C. Polypeptides were extracted once with phenol:chloroform:isoamyl alcohol mixture (25:24:1) and once with chloroform:isoamyl alcohol (24:1). DNA products were analyzed by 2% agarose gel electrophoresis.

### Gel Electrophoresis and Analysis of Dissolution and Resolution Products.

Following the addition of DNA loading dye, samples were loaded onto a 2% agarose gel in TBE buffer (Tris-boric acid-EDTA) and run at 5.5 V/cm for 2 h in TBE at 4 °C. The gel was stained with ethidium bromide for 3 h (with shaking) or overnight (without shaking), and destained for 30 min in water. Images were acquired using a BioRad Gel Doc XR+ system and band intensity was measured using the Gel Analyzer tool in Fiji (39).

## Supplementary Material

Supplementary File

## Data Availability

All study data are included in the article and/or *SI Appendix*.
