# Supplementary material for: Generation of double Holliday junction DNAs and their dissolution/resolution within a chromatin context
Source: Proc Natl Acad Sci U S A. 2022 Apr 22;119(18):e2123420119. doi: 10.1073/pnas.2123420119 (PMC9170140; doi:10.1073/pnas.2123420119)
Supplement: Supplementary File [file pnas.2123420119.sapp.pdf]

PNAS
www.pnas.org

Supplementary information for

Generation of double Holliday junction DNAs and their resolution/dissolution within a chromatin context

Han N. Ho and Stephen C. West

DNA Recombination and Repair Laboratory, The Francis Crick Institute,

1 Midland Road, London NW1 1AT, UK

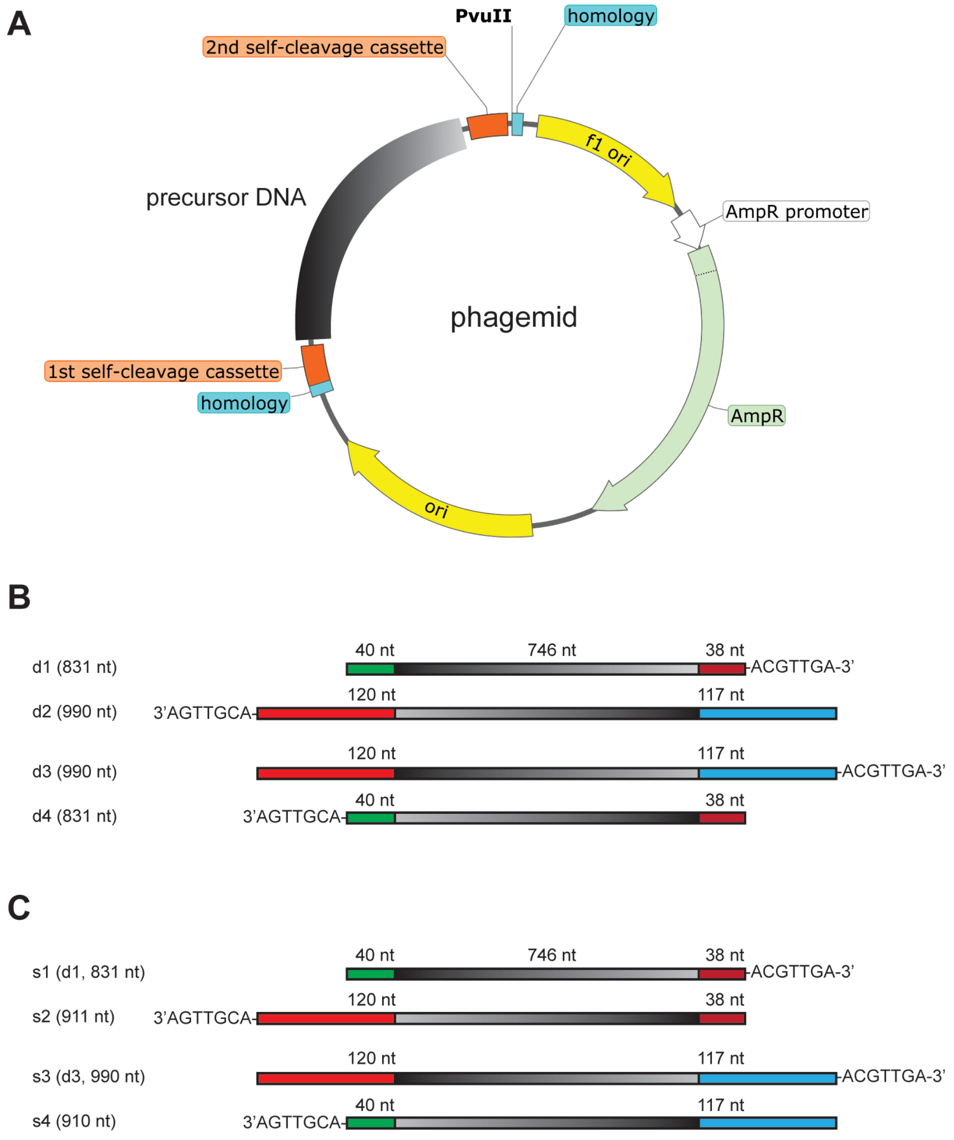

Fig. S1. Phagemids and precursor ssDNAs for the generation of dHJ and sHJ DNAs. (A) Diagram illustrating key elements of the phagemid used in this study: *f1* origin of replication, *AmpR* antibiotic resistance cassette, *ori* origin of replication, and synthetic DNA comprised of homologous sequences (to phagemid backbone, shown in cyan), DNAzyme self-cleavage cassettes (shown in orange) and precursor DNA (black-to-grey shade). The black-to-grey shading is in sync with the orientation of *f1*, which dictates the direction of rolling circle DNA synthesis, hence, the strand being produced and packed into viral particles.
(B-C) Diagrams illustrating the design principles of precursor ssDNAs d1-d4 (B) and s1-s4 (C). The 746-bp region of homology is at the center, and black-to-grey shading indicates the 5' to 3' direction. Blocks of the same color represent complementary sequences between different precursor ssDNAs. The 7-nt ssDNA tail at the 3' end (5'-ACGTTGA-3') remains single-stranded in the final products.

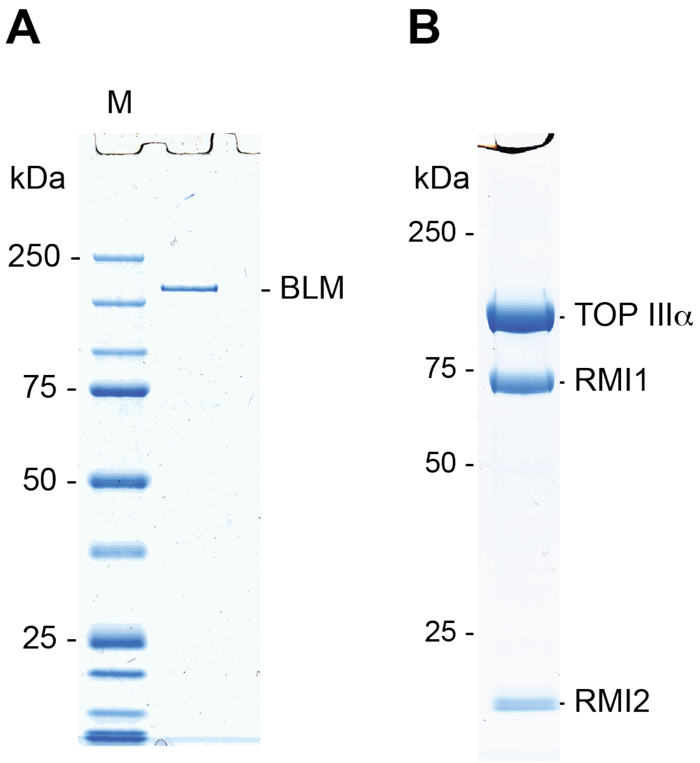

Fig. S2. SDS-PAGE analysis of recombinant (A) BLM and (B) TRR complex. Proteins were visualized by staining with Coomassie blue. M: protein size markers.

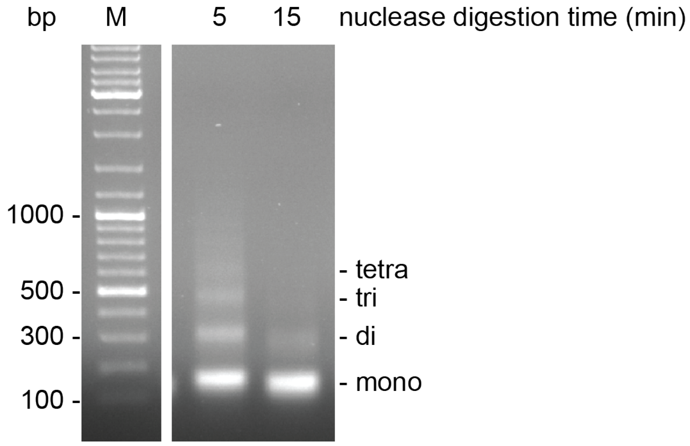

Fig. S3. Micrococcal nuclease digestion of chromatinized dHJ DNA. Reactions contained chromatinized dHJ DNA (220 ng) with micrococcal nuclease (300 units), and were incubated at 37°C for the indicated times. Products were resolved by 1.3% agarose gel electrophoresis and visualized by ethidium bromide staining. Band pattern represents DNA in mononucleosomes, dinucleosomes, trinucleosomes and tetranucleosomes. M: DNA size markers (in bp).

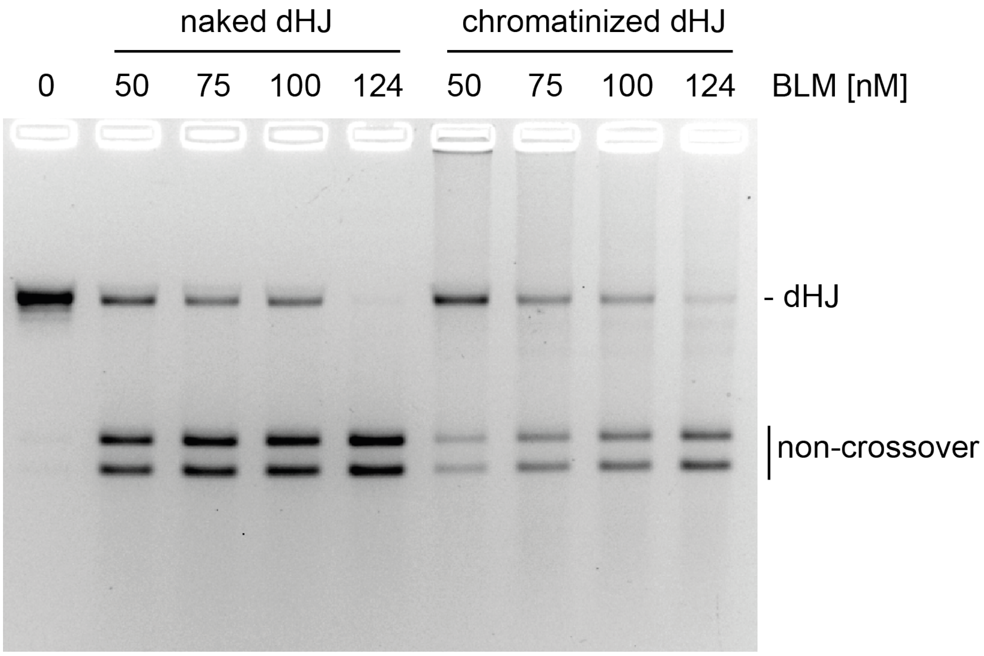

Fig. S4. Dissolution of naked or chromatinized dHJ by varying concentrations of BLM. Dissolution reactions contained naked or chromatinized dHJ (2.6 nM), TRR (320 nM) and the indicated concentrations of BLM. Reactions were incubated at 37°C for 1 hour. Products were resolved by 2% agarose gel electrophoresis and visualized by ethidium bromide staining.

Table S1. Plasmid list.

Plasmid name	Usage	Source
pdHJ1 (psHJ1)	Generation of dHJ and sHJ	This study
pdHJ2	Generation of dHJ	This study
pdHJ3 (psHJ3)	Generation of dHJ and sHJ	This study
pdHJ4	Generation of dHJ	This study
psHJ2	Generation of sHJ	This study
psHJ4	Generation of sHJ	This study
HP4_M13 (Helper plasmid)	Production of circular ssDNA	(1)
pFL-TRR	Purification of the TRR complex from *Sf*9	This study
pFL-T^{Y362F}RR	Purification of the T^{Y362F}RR complex from *Sf*9	This study
pFB-$_{MBP}$BLM$_{His}$	Purification of BLM helicase	(2)
pYes-GEN1-3xFlag-His10	Purification of GEN1	(3)
pFastBACDual-$_{V5}$SLX1-$_{StrepII}$SLX4	Purification of the SMX complex	(4)
pFL/pSPL-$_{V5}$SLX1-$_{Strep}$SLX4-MUS81-$_{FLAG}$EME1	Purification of the SMX complex	(5)
pFL-XPF-$_{His6}$ERCC1	Purification of the SMX complex	(6)
pCDFduet.H2A-H2B	Purification of yeast histone	(7)
pETduet.H3-H4	Purification of yeast histone	(7)
pCFK1	Purification of yeast Nap1	(8)

Table S2. Sequence of pdHJ1/psHJ1 (3486 bp). Lower case: backbone, upper case: precursor DNA (green, black and dark red correspond to color blocks in d1/s1 schematic diagrams in Fig. S1B-C, bold: 7-nt ssDNA tail), underlined: self-cleavage DNAzyme cassettes. The sites of cleavage are highlighted in yellow.

ctggcgtaatagcgaagaggcccgcaccgatcgcccttcccaacagttgcgcagcctgaatggcgaatgggacgcgccctgtagcggcgcattaagcgcggcgggtgtggtggttacgcgcagcgtgaccgctacacttg
ccagcgccctagcgcccgctcctttcgctttcttcccttcctttctcgccacgttcgccggctttccccgtcaagctctaaatcgggggctccctttagggttccgatttagtgctttacggcacctcgaccccaaaaaacttgatt
agggtgatggttcacgtagtgggccatcgccctgatagacggtttttcgccctttgacgttggagtccacgttctttaatagtggactcttgttccaaactggaacaacactcaaccctatctcggtctattcttttgatttataa
gggattttgccgatttcggcctattggttaaaaaatgagctgatttaacaaaaatttaacgcgaattttaacaaaatattaacgcttacaatttaggtggcacttttcggggaaatgtgcgcggaacccctatttgtttatttttc
taaatacattcaaatatgtatccgctcatgagacaataaccctgataaatgcttcaataatattgaaaaaggaagagtatgagtattcaacatttccgtgtcgcccttattcccttttttgcggcattttgccttcctgtttttgct
cacccagaaacgctggtgaaagtaaaagatgctgaagatcagttgggtgcacgagtgggttacatcgaactggatctcaacagcggtaagatccttgagagttttcgccccgaagaacgttttccaatgatgagcacttttt
aaagttctgctatgtggcgcggtattatcccgtattgacgccgggcaagagcaactcggtcgccgcatacactattctcagaatgacttggttgagtactcaccagtcacagaaaagcatcttacggatggcatgacagtaa
gagaattatgcagtgctgccataaccatgagtgataacactgcggccaacttacttctgacaacgatcggaggaccgaaggagctaaccgcttttttgcacaacatgggggatcatgtaactcgccttgatcgttgggaacc
ggagctgaatgaagccataccaaacgacgagcgtgacaccacgatgcctgtagcaatggcaacaacgttgcgcaaactattaactggcgaactacttactctagcttcccggcaacaattaatagactggatggaggcgg
ataaagttgcaggaccacttctgcgctcggcccttccggctggctggtttattgctgataaatctggagccggtgagcgtgggtctcgcggtatcattgcagcactggggccagatggtaagccctcccgtatcgtagttatct
acacgacggggagtcaggcaactatggatgaacgaaatagacagatcgctgagataggtgcctcactgattaagcattggtaactgtcagaccaagtttactcatatatactttagattgatttaaaacttcatttttaattt
aaaaggatctaggtgaagatcctttttgataatctcatgaccaaaatcccttaacgtgagttttcgttccactgagcgtcagaccccgtagaaaagatcaaaggatcttcttgagatcctttttttctgcgcgtaatctgctgctt
gcaaacaaaaaaaccaccgctaccagcggtggtttgtttgccggatcaagagctaccaactctttttccgaaggtaactggcttcagcagagcgcagataccaaatactgtccttctagtgtagccgtagttaggccaccac
ttcaagaactctgtagcaccgcctacatacctcgctctgctaatcctgttaccagtggctgctgccagtggcgataagtcgtgtcttaccgggttggactcaagacgatagttaccggataaggcgcagcggtcgggctgaac
ggggggttcgtgcacacagcccagcttggagcgaacgacctacaccgaactgagatacctacagcgtgagctatgagaaagcgccacgcttcccgaagggagaaaggcggacaggtatccggtaagcggcagggtcgg
aacaggagagcgcacgagggagcttccagggggaaacgcctggtatctttatagtcctgtcgggtttcgccacctctgacttgagcgtcgatttttgtgatgctcgtcaggggggcggagcctatggaaaacgccagcaac
gcggcctttttacggttcctggccttttgctggccttttgctcacatgttctttcctgcgttatcccctgattctgtggataaccgtattaccgcctttgagtgagctgataccgctcgccgcagccgaacgaccgagcgcagcga
gtcagtgagcgaggaagcgattgcacgttgaagcgttacctgttaggtaacgtagttgagctgtgcaatctatttttagaggtcctagttgagctgtcacagaatgtgacgttgaAGGGACCTCTGTAATCGACTC
GCTGATAGGCTGTTCGAGAATGTATAGAGATGAGTATGTCATTCAAGCTCTAGAGTTGTAGTCCGCTGTATAAGCCCCCGATGACCGTCTTTACCCGAATCTATCGTCCT
GAAGGGTTCAACGCTACGCCAGCTCAGTAGAATACTCAGCCCAGTTACGCTAAAATGTCTCAGGGATGGACTGCGATCACGTTTTGTGGGTATCCGGAGTGCACCTCAA
ATCCCCGACAGCTTGCTCTCGCCTGGTAACAGTAACCGATAGGATCTCGGCACTGAGAACACCTTCAGGTCCCTCTGGTGACTGGGTAGTTGGACTGTGCCCTTGGATA
GAATAGCAAGAGCCTGACTCTCTATTGTTGACACGACGACTGTGGGAGACCTCAGCGTGCAGACATCAGATCGAGTAATACTAAGGTGGGATAACTCCGTAACTGACT
ACGTTGCCTTCTCCACATATGGAGCTGGATAGAACAGCCCTGGTGTGCACCATCAGCAACCCTATACGTGGCTTTTCGTGAGGCTTTGGCACGGAGTAGGCGATGTCTT
CCACTATTCGAATGGCCGTTCGTTAATACTTGTTGCGTAGCTAGCACTATATTTGTCTCTTTGCCGACTGATGTCGACAAGCACACATAGCCATTTATCGGAGCGCCTCGG
AATACGGTATTGCATGAGTAGAGTTGACTAAGAGCCATGAGCAGGGCCTCGTGAGACCATTGCGAATACCAGGTGTCCTGTAAGAGCGAACCCATACGACTGCTAGGA
AAGCGAACCGGTGGTGAATTTAATCTTCTGGTGTAGCCT**ACGTTGA**agcgttacctgttaggtaacgtagttgagctgtaggctagatttttgctcacattagttgagctgtcacagaatgtgacgttga
agatgtgagcccctttcgccag

Table S3. Sequence of pdHJ2 (3645 bp). Lower case: backbone, upper case: precursor DNA (blue, black and red correspond to color blocks in d2 schematic diagram in Fig. S1B, bold: 7-nt ssDNA tail), underlined: self-cleavage DNAzyme cassettes. The sites of cleavage are highlighted in yellow.

ctggcgtaatagcgaagaggcccgcaccgatcgcccttcccaacagttgcgcagcctgaatggcgaatgggacgcgccctgtagcggcgcattaagcgcggcgggtgtggtggttacgcgcagcgtgaccgctacacttg
ccagcgccctagcgcccgctcctttcgctttcttcccttcctttctcgccacgttcgccggctttccccgtcaagctctaaatcgggggctccctttagggttccgatttagtgctttacggcacctcgaccccaaaaaacttgatt
agggtgatggttcacgtagtgggccatcgccctgatagacggtttttcgccctttgacgttggagtccacgttctttaatagtggactcttgttccaaactggaacaacactcaaccctatctcggtctattcttttgatttataa
gggattttgccgatttcggcctattggttaaaaaatgagctgatttaacaaaaatttaacgcgaattttaacaaaatattaacgcttacaatttaggtggcacttttcggggaaatgtgcgcggaacccctatttgtttatttttc
taaatacattcaaatatgtatccgctcatgagacaataaccctgataaatgcttcaataatattgaaaaaggaagagtatgagtattcaacatttccgtgtcgcccttattcccttttttgcggcattttgccttcctgtttttgct
cacccagaaacgctggtgaaagtaaaagatgctgaagatcagttgggtgcacgagtgggttacatcgaactggatctcaacagcggtaagatccttgagagttttcgccccgaagaacgttttccaatgatgagcacttttt
aaagttctgctatgtggcgcggtattatcccgtattgacgccgggcaagagcaactcggtcgccgcatacactattctcagaatgacttggttgagtactcaccagtcacagaaaagcatcttacggatggcatgacagtaa
gagaattatgcagtgctgccataaccatgagtgataacactgcggccaacttacttctgacaacgatcggaggaccgaaggagctaaccgcttttttgcacaacatgggggatcatgtaactcgccttgatcgttgggaacc
ggagctgaatgaagccataccaaacgacgagcgtgacaccacgatgcctgtagcaatggcaacaacgttgcgcaaactattaactggcgaactacttactctagcttcccggcaacaattaatagactggatggaggcgg
ataaagttgcaggaccacttctgcgctcggcccttccggctggctggtttattgctgataaatctggagccggtgagcgtgggtctcgcggtatcattgcagcactggggccagatggtaagccctcccgtatcgtagttatct
acacgacggggagtcaggcaactatggatgaacgaaatagacagatcgctgagataggtgcctcactgattaagcattggtaactgtcagaccaagtttactcatatatactttagattgatttaaaacttcatttttaattt
aaaaggatctaggtgaagatcctttttgataatctcatgaccaaaatcccttaacgtgagttttcgttccactgagcgtcagaccccgtagaaaagatcaaaggatcttcttgagatcctttttttctgcgcgtaatctgctgctt
gcaaacaaaaaaaccaccgctaccagcggtggtttgtttgccggatcaagagctaccaactctttttccgaaggtaactggcttcagcagagcgcagataccaaatactgtccttctagtgtagccgtagttaggccaccac
ttcaagaactctgtagcaccgcctacatacctcgctctgctaatcctgttaccagtggctgctgccagtggcgataagtcgtgtcttaccgggttggactcaagacgatagttaccggataaggcgcagcggtcgggctgaac
ggggggttcgtgcacacagcccagcttggagcgaacgacctacaccgaactgagatacctacagcgtgagctatgagaaagcgccacgcttcccgaagggagaaaggcggacaggtatccggtaagcggcagggtcgg
aacaggagagcgcacgagggagcttccagggggaaacgcctggtatctttatagtcctgtcgggtttcgccacctctgacttgagcgtcgatttttgtgatgctcgtcaggggggcggagcctatggaaaacgccagcaac
gcggcctttttacggttcctggccttttgctggccttttgctcacatgttctttcctgcgttatcccctgattctgtggataaccgtattaccgcctttgagtgagctgataccgctcgccgcagccgaacgaccgagcgcagcga
gtcagtgagcgaggaagcgattgcacgttgaagcgttacctgttaggtaacgtagttgagctgtgcaatctatttttaacaacgatagttgagctgtcacagaatgtgacgttgaAGTCGTTGTTGGACTCATTCAA
CCATGGGCTCCAGGGGCGGAAGAGCAGGCAAGCTGCGGACACAAATTTCTCGACAGTAGGGATATGTTTCACTACGTGTGGCGTCCTGGACAGGGTTCCTAGCAGTC
GTATGGGTTCGCTCTTACAGGACACCTGGTATTCGCAATGGTCTCACGAGGCCCTGCTCATGGCTCTTAGTCAACTCTACTCATGCAATACCGTATTCCGAGGCGCTCCG
ATAAATGGCTATGTGTGCTTGTCGACATCAGTCGGCAAAGAGACAAATATAGTGCTAGCTACGCAACAAGTATTAACGAACGGCCATTCGAATAGTGGAAGACATCGC
CTACTCCGTGCCAAAGCCTCACGAAAAGCCACGTATAGGGTTGCTGATGGTGCACACCAGGGCTGTTCTATCCAGCTCCATATGTGGAGAAGGCAACGTAGTCAGTTAC
GGAGTTATCCCACCTTAGTATTACTCGATCTGATGTCTGCACGCTGAGGTCTCCCACAGTCGTCGTGTCAACAATAGAGAGTCAGGCTCTTGCTATTCTATCCAAGGGCA
CAGTCCAACTACCCAGTCACCAGAGGGACCTGAAGGTGTTCTCAGTGCCGAGATCCTATCGGTTACTGTTACCAGGCGAGAGCAAGCTGTCGGGGATTTGAGGTGCAC
TCCGGATACCCACAAAACGTGATCGCAGTCCATCCCTGAGACATTTTAGCGTAACTGGGCTGAGTATTCTACTGAGCTGGCGTAGCGTTGAACCCTTCAGGACGATAGA
TTCGGGTAAAGACGGTCATCGGGGGCTTATACAGCGGACTACAACTCTAGAGCTTGAATGACATACTCATCTCTATACATCGGATGTGCGGGTCGAAGAGGTCGACAG
GGTAGGCCTAATCGGGTTGCAGTTAGTGACGGGCAAGCAGATTTATTATAGCGCGAAGATCCTGCTGACTCCTGTTAAACGCATTGGCTGCT**ACGTTGA**agcgttacctgtt
aggtaacgtagttgagctgtagcagcgatttttgctcacattagttgagctgtcacagaatgtgacgttgaagatgtgagccccttcgccag

Table S4. Sequence of pdHJ3/psHJ3 (3645 bp). Lower case: backbone, upper case: precursor DNA (red, black and blue correspond to color blocks in d3/s3 schematic diagrams in Fig. S1B-C, bold: 7-nt ssDNA tail), underlined: self-cleavage DNAzyme cassettes. The sites of cleavage are highlighted in yellow.

ctggcgtaatagcgaagaggcccgcaccgatcgcccttcccaacagttgcgcagcctgaatggcgaatgggacgcgccctgtagcggcgcattaagcgcggcgggtgtggtggttacgcgcagcgtgaccgctacacttg
ccagcgccctagcgcccgctcctttcgctttcttcccttcctttctcgccacgttcgccggctttccccgtcaagctctaaatcgggggctccctttagggttccgatttagtgctttacggcacctcgaccccaaaaaacttgatt
agggtgatggttcacgtagtgggccatcgccctgatagacggtttttcgccctttgacgttggagtccacgttctttaatagtggactcttgttccaaactggaacaacactcaaccctatctcggtctattcttttgatttataa
gggattttgccgatttcggcctattggttaaaaaatgagctgatttaacaaaaatttaacgcgaattttaacaaaatattaacgcttacaatttaggtggcacttttcggggaaatgtgcgcggaacccctatttgtttatttttc
taaatacattcaaatatgtatccgctcatgagacaataaccctgataaatgcttcaataatattgaaaaaggaagagtatgagtattcaacatttccgtgtcgcccttattcccttttttgcggcattttgccttcctgtttttgct
cacccagaaacgctggtgaaagtaaaagatgctgaagatcagttgggtgcacgagtgggttacatcgaactggatctcaacagcggtaagatccttgagagttttcgccccgaagaacgttttccaatgatgagcacttttt
aaagttctgctatgtggcgcggtattatcccgtattgacgccgggcaagagcaactcggtcgccgcatacactattctcagaatgacttggttgagtactcaccagtcacagaaaagcatcttacggatggcatgacagtaa
gagaattatgcagtgctgccataaccatgagtgataacactgcggccaacttacttctgacaacgatcggaggaccgaaggagctaaccgcttttttgcacaacatgggggatcatgtaactcgccttgatcgttgggaacc
ggagctgaatgaagccataccaaacgacgagcgtgacaccacgatgcctgtagcaatggcaacaacgttgcgcaaactattaactggcgaactacttactctagcttcccggcaacaattaatagactggatggaggcgg
ataaagttgcaggaccacttctgcgctcggcccttccggctggctggtttattgctgataaatctggagccggtgagcgtgggtctcgcggtatcattgcagcactggggccagatggtaagccctcccgtatcgtagttatct
acacgacggggagtcaggcaactatggatgaacgaaatagacagatcgctgagataggtgcctcactgattaagcattggtaactgtcagaccaagtttactcatatatactttagattgatttaaaacttcatttttaattt
aaaaggatctaggtgaagatcctttttgataatctcatgaccaaaatcccttaacgtgagttttcgttccactgagcgtcagaccccgtagaaaagatcaaaggatcttcttgagatcctttttttctgcgcgtaatctgctgctt
gcaaacaaaaaaaccaccgctaccagcggtggtttgtttgccggatcaagagctaccaactctttttccgaaggtaactggcttcagcagagcgcagataccaaatactgtccttctagtgtagccgtagttaggccaccac
ttcaagaactctgtagcaccgcctacatacctcgctctgctaatcctgttaccagtggctgctgccagtggcgataagtcgtgtcttaccgggttggactcaagacgatagttaccggataaggcgcagcggtcgggctgaac
ggggggttcgtgcacacagcccagcttggagcgaacgacctacaccgaactgagatacctacagcgtgagctatgagaaagcgccacgcttcccgaagggagaaaggcggacaggtatccggtaagcggcagggtcgg
aacaggagagcgcacgagggagcttccagggggaaacgcctggtatctttatagtcctgtcgggtttcgccacctctgacttgagcgtcgatttttgtgatgctcgtcaggggggcggagcctatggaaaacgccagcaac
gcggcctttttacggttcctggccttttgctggccttttgctcacatgttctttcctgcgttatcccctgattctgtggataaccgtattaccgcctttgagtgagctgataccgctcgccgcagccgaacgaccgagcgcagcga
gtcagtgagcgaggaagcgattgcacgttgaagcgttacctgttaggtaacgtagttgagctgtgcaatctatttttattggctgtagttgagctgtcacagaatgtgacgttgaAGCAGCCAATGCGTTTAACAGG
AGTCAGCAGGATCTTCGCGCTATAATAAATCTGCTTGCCCGTCACTAACTGCAACCCGATTAGGCCTACCCTGTCGACCTCTTCGACCCGCACATCCGATGTATAGAGAT
GAGTATGTCATTCAAGCTCTAGAGTTGTAGTCCGCTGTATAAGCCCCCGATGACCGTCTTTACCCGAATCTATCGTCCTGAAGGGTTCAACGCTACGCCAGCTCAGTAGA
ATACTCAGCCCAGTTACGCTAAAATGTCTCAGGGATGGACTGCGATCACGTTTTGTGGGTATCCGGAGTGCACCTCAAATCCCCGACAGCTTGCTCTCGCCTGGTAACA
GTAACCGATAGGATCTCGGCACTGAGAACACCTTCAGGTCCCTCTGGTGACTGGGTAGTTGGACTGTGCCCTTGGATAGAATAGCAAGAGCCTGACTCTCTATTGTTGA
CACGACGACTGTGGGAGACCTCAGCGTGCAGACATCAGATCGAGTAATACTAAGGTGGGATAACTCCGTAACTGACTACGTTGCCTTCTCCACATATGGAGCTGGATA
GAACAGCCCTGGTGTGCACCATCAGCAACCCTATACGTGGCTTTTCGTGAGGCTTTGGCACGGAGTAGGCGATGTCTTCCACTATTCGAATGGCCGTTCGTTAATACTTG
TTGCGTAGCTAGCACTATATTTGTCTCTTTGCCGACTGATGTCGACAAGCACACATAGCCATTTATCGGAGCGCCTCGGAATACGGTATTGCATGAGTAGAGTTGACTAA
GAGCCATGAGCAGGGCCTCGTGAGACCATTGCGAATACCAGGTGTCCTGTAAGAGCGAACCCATACGACTGCTAGGAACCCTGTCCAGGACGCCACACGTAGTGAAAC
ATATCCCTACTGTCGAGAAATTTGTGTCCGCAGCTTGCCTGCTCTTCCGCCCCTGGAGCCCATGGTTGAATGAGTCCAACAACGACT**ACGTTGA**agcgttacctgttaggtaacg
tagttgagctgtagtcgtgatttttgctcacattagttgagctgtcacagaatgtgacgttgaagatgtgagccccttcgccag

Table S5. Sequence of pdHJ4 (3486 bp). Lower case: backbone, upper case: precursor DNA (dark red, black and green correspond to color blocks in d4 schematic diagrams in Fig. S1B, bold: 7-nt ssDNA tail), underlined: self-cleavage DNAzyme cassettes. The sites of cleavage are highlighted in yellow.

ctggcgtaatagcgaagaggcccgcaccgatcgcccttcccaacagttgcgcagcctgaatggcgaatgggacgcgccctgtagcggcgcattaagcgcggcgggtgtggtggttacgcgcagcgtgaccgctacacttg
ccagcgccctagcgcccgctcctttcgctttcttcccttcctttctcgccacgttcgccggctttccccgtcaagctctaaatcgggggctccctttagggttccgatttagtgctttacggcacctcgaccccaaaaaacttgatt
agggtgatggttcacgtagtgggccatcgccctgatagacggtttttcgccctttgacgttggagtccacgttctttaatagtggactcttgttccaaactggaacaacactcaaccctatctcggtctattcttttgatttataa
gggattttgccgatttcggcctattggttaaaaaatgagctgatttaacaaaaatttaacgcgaattttaacaaaatattaacgcttacaatttaggtggcacttttcggggaaatgtgcgcggaacccctatttgtttatttttc
taaatacattcaaatatgtatccgctcatgagacaataaccctgataaatgcttcaataatattgaaaaaggaagagtatgagtattcaacatttccgtgtcgcccttattcccttttttgcggcattttgccttcctgtttttgct
cacccagaaacgctggtgaaagtaaaagatgctgaagatcagttgggtgcacgagtgggttacatcgaactggatctcaacagcggtaagatccttgagagttttcgccccgaagaacgttttccaatgatgagcacttttt
aaagttctgctatgtggcgcggtattatcccgtattgacgccgggcaagagcaactcggtcgccgcatacactattctcagaatgacttggttgagtactcaccagtcacagaaaagcatcttacggatggcatgacagtaa
gagaattatgcagtgctgccataaccatgagtgataacactgcggccaacttacttctgacaacgatcggaggaccgaaggagctaaccgcttttttgcacaacatgggggatcatgtaactcgccttgatcgttgggaacc
ggagctgaatgaagccataccaaacgacgagcgtgacaccacgatgcctgtagcaatggcaacaacgttgcgcaaactattaactggcgaactacttactctagcttcccggcaacaattaatagactggatggaggcgg
ataaagttgcaggaccacttctgcgctcggcccttccggctggctggtttattgctgataaatctggagccggtgagcgtgggtctcgcggtatcattgcagcactggggccagatggtaagccctcccgtatcgtagttatct
acacgacggggagtcaggcaactatggatgaacgaaatagacagatcgctgagataggtgcctcactgattaagcattggtaactgtcagaccaagtttactcatatatactttagattgatttaaaacttcatttttaattt
aaaaggatctaggtgaagatcctttttgataatctcatgaccaaaatcccttaacgtgagttttcgttccactgagcgtcagaccccgtagaaaagatcaaaggatcttcttgagatcctttttttctgcgcgtaatctgctgctt
gcaaacaaaaaaaccaccgctaccagcggtggtttgtttgccggatcaagagctaccaactctttttccgaaggtaactggcttcagcagagcgcagataccaaatactgtccttctagtgtagccgtagttaggccaccac
ttcaagaactctgtagcaccgcctacatacctcgctctgctaatcctgttaccagtggctgctgccagtggcgataagtcgtgtcttaccgggttggactcaagacgatagttaccggataaggcgcagcggtcgggctgaac
ggggggttcgtgcacacagcccagcttggagcgaacgacctacaccgaactgagatacctacagcgtgagctatgagaaagcgccacgcttcccgaagggagaaaggcggacaggtatccggtaagcggcagggtcgg
aacaggagagcgcacgagggagcttccagggggaaacgcctggtatctttatagtcctgtcgggtttcgccacctctgacttgagcgtcgatttttgtgatgctcgtcaggggggcggagcctatggaaaaacgccagcaac
gcggcctttttacggttcctggccttttgctggccttttgctcacatgttctttcctgcgttatcccctgattctgtggataaccgtattaccgcctttgagtgagctgataccgctcgccgcagccgaacgaccgagcgcagcga
gtcagtgagcgaggaagcgattgcacgttgaagcgttacctgttaggtaacgtagttgagctgtgcaatctatttttggtgtagctagttgagctgtcacagaatgtgacgttgaAGGCTACACCAGAAGATTAAA
TTCACCACCGGTTCGCTTTCCTAGCAGTCGTATGGGTTCGCTCTTACAGGACACCTGGTATTCGCAATGGTCTCACGAGGCCCTGCTCATGGCTCTTAGTCAACTCTACTC
ATGCAATACCGTATTCCGAGGCGCTCCGATAAATGGCTATGTGTGCTTGTCGACATCAGTCGGCAAAGAGACAAATATAGTGCTAGCTACGCAACAAGTATTAACGAAC
GGCCATTCGAATAGTGGAAGACATCGCCTACTCCGTGCCAAAGCCTCACGAAAAGCCACGTATAGGGTTGCTGATGGTGCACACCAGGGCTGTTCTATCCAGCTCCATA
TGTGGAGAAGGCAACGTAGTCAGTTACGGAGTTATCCCACCTTAGTATTACTCGATCTGATGTCTGCACGCTGAGGTCTCCCACAGTCGTCGTGTCAACAATAGAGAGT
CAGGCTCTTGCTATTCTATCCAAGGGCACAGTCCAACTACCCAGTCACCAGAGGGACCTGAAGGTGTTCTCAGTGCCGAGATCCTATCGGTTACTGTTACCAGGCGAGA
GCAAGCTGTCGGGGATTTGAGGTGCACTCCGGATACCCACAAAACGTGATCGCAGTCCATCCCTGAGACATTTTAGCGTAACTGGGCTGAGTATTCTACTGAGCTGGCG
TAGCGTTGAACCCTTCAGGACGATAGATTCGGGTAAAGACGGTCATCGGGGGCTTATACAGCGGACTACAACTCTAGAGCTTGAATGACATACTCATCTCTATACATTC
TCGAACAGCCTATCAGCGAGTCGATTACAGAGGTCCCT**ACGTTGA**agcgttacctgttaggtaacgtagttgagctgtagggacgatttttgctcacattagttgagctgtcacagaatgtgacgttgaa
gatgtgagccccctttcgccag

Table S6. Sequence of psHJ2 (3566 bp). Lower case: backbone, upper case: precursor DNA (dark red, black and red correspond to color blocks in s2 schematic diagrams in Fig. S1C, bold: 7-nt ssDNA tail), underlined: self-cleavage DNAzyme cassettes. The sites of cleavage are highlighted in yellow.

ctggcgtaatagcgaagaggcccgcaccgatcgcccttcccaacagttgcgcagcctgaatggcgaatgggacgcgccctgtagcggcgcattaagcgcggcgggtgtggtggttacgcgcagcgtgaccgctacacttg
ccagcgccctagcgcccgctcctttcgctttcttcccttcctttctcgccacgttcgccggctttccccgtcaagctctaaatcgggggctccctttagggttccgatttagtgctttacggcacctcgaccccaaaaaacttgatt
agggtgatggttcacgtagtgggccatcgccctgatagacggtttttcgccctttgacgttggagtccacgttctttaatagtggactcttgttccaaactggaacaacactcaaccctatctcggtctattcttttgatttataa
gggattttgccgatttcggcctattggttaaaaaatgagctgatttaacaaaaatttaacgcgaattttaacaaaatattaacgcttacaatttaggtggcacttttcggggaaatgtgcgcggaacccctatttgtttatttttc
taaatacattcaaatatgtatccgctcatgagacaataaccctgataaatgcttcaataatattgaaaaaggaagagtatgagtattcaacatttccgtgtcgcccttattcccttttttgcggcattttgccttcctgtttttgct
cacccagaaacgctggtgaaagtaaaagatgctgaagatcagttgggtgcacgagtgggttacatcgaactggatctcaacagcggtaagatccttgagagttttcgccccgaagaacgttttccaatgatgagcacttttt
aaagttctgctatgtggcgcggtattatcccgtattgacgccgggcaagagcaactcggtcgccgcatacactattctcagaatgacttggttgagtactcaccagtcacagaaaagcatcttacggatggcatgacagtaa
gagaattatgcagtgctgccataaccatgagtgataacactgcggccaacttacttctgacaacgatcggaggaccgaaggagctaaccgcttttttgcacaacatgggggatcatgtaactcgccttgatcgttgggaacc
ggagctgaatgaagccataccaaacgacgagcgtgacaccacgatgcctgtagcaatggcaacaacgttgcgcaaactattaactggcgaactacttactctagcttcccggcaacaattaatagactggatggaggcgg
ataaagttgcaggaccacttctgcgctcggcccttccggctggctggtttattgctgataaatctggagccggtgagcgtgggtctcgcggtatcattgcagcactggggccagatggtaagccctcccgtatcgtagttatct
acacgacggggagtcaggcaactatggatgaacgaaatagacagatcgctgagataggtgcctcactgattaagcattggtaactgtcagaccaagtttactcatatatactttagattgatttaaaacttcatttttaattt
aaaaggatctaggtgaagatcctttttgataatctcatgaccaaaatcccttaacgtgagttttcgttccactgagcgtcagaccccgtagaaaagatcaaaggatcttcttgagatcctttttttctgcgcgtaatctgctgctt
gcaaacaaaaaaaccaccgctaccagcggtggtttgtttgccggatcaagagctaccaactctttttccgaaggtaactggcttcagcagagcgcagataccaaatactgtccttctagtgtagccgtagttaggccaccac
ttcaagaactctgtagcaccgcctacatacctcgctctgctaatcctgttaccagtggctgctgccagtggcgataagtcgtgtcttaccgggttggactcaagacgatagttaccggataaggcgcagcggtcgggctgaac
ggggggttcgtgcacacagcccagcttggagcgaacgacctacaccgaactgagatacctacagcgtgagctatgagaaagcgccacgcttcccgaagggagaaaggcggacaggtatccggtaagcggcagggtcgg
aacaggagagcgcacgagggagcttccagggggaaacgcctggtatctttatagtcctgtcgggtttcgccacctctgacttgagcgtcgatttttgtgatgctcgtcaggggggcggagcctatggaaaacgccagcaac
gcggcctttttacggttcctggccttttgctggccttttgctcacatgttctttcctgcgttatcccctgattctgtggataaccgtattaccgcctttgagtgagctgataccgctcgccgcagccgaacgaccgagcgcagcga
gtcagtgagcgaggaagcgattgcacgttgaagcgttacctgttaggtaacgtagttgagctgtgcaatctatttttggtgtagctagttgagctgtcacagaatgtgacgttgaAGGCTACACCAGAAGATTAAA
TTCACCACCGGTTCGCTTTCCTAGCAGTCGTATGGGTTCGCTCTTACAGGACACCTGGTATTCGCAATGGTCTCACGAGGCCCTGCTCATGGCTCTTAGTCAACTCTACTC
ATGCAATACCGTATTCCGAGGCGCTCCGATAAATGGCTATGTGTGCTTGTCGACATCAGTCGGCAAAGAGACAAATATAGTGCTAGCTACGCAACAAGTATTAACGAAC
GGCCATTCGAATAGTGGAAGACATCGCCTACTCCGTGCCAAAGCCTCACGAAAAGCCACGTATAGGGTTGCTGATGGTGCACACCAGGGCTGTTCTATCCAGCTCCATA
TGTGGAGAAGGCAACGTAGTCAGTTACGGAGTTATCCCACCTTAGTATTACTCGATCTGATGTCTGCACGCTGAGGTCTCCCACAGTCGTCGTGTCAACAATAGAGAGT
CAGGCTCTTGCTATTCTATCCAAGGGCACAGTCCAACTACCCAGTCACCAGAGGGACCTGAAGGTGTTCTCAGTGCCGAGATCCTATCGGTTACTGTTACCAGGCGAGA
GCAAGCTGTCGGGGATTTGAGGTGCACTCCGGATACCCACAAAACGTGATCGCAGTCCATCCCTGAGACATTTTAGCGTAACTGGGCTGAGTATTCTACTGAGCTGGCG
TAGCGTTGAACCCTTCAGGACGATAGATTCGGGTAAAGACGGTCATCGGGGGCTTATACAGCGGACTACAACTCTAGAGCTTGAATGACATACTCATCTCTATACATCG
GATGTGCGGGTCGAAGAGGTCGACAGGGTAGGCCTAATCGGGTTGCAGTTAGTGACGGGCAAGCAGATTTATTATAGCGCGAAGATCCTGCTGACTCCTGTTAAACGC
ATTGGCTGCT**ACGTTGA**agcgttacctgttaggtaacgtagttgagctgtagcagcgatttttgctcacattagttgagctgtcacagaatgtgacgttgaagatgtgagccccttttcgccag

Table S7. Sequence of psHJ4 (3565 bp). Lower case: backbone, upper case: precursor DNA (blue, black and green correspond to color blocks in s4 schematic diagrams in Fig. S1C, bold: 7-nt ssDNA tail), underlined: self-cleavage DNAzyme cassettes. The sites of cleavage are highlighted in yellow.

ctggcgtaatagcgaagaggcccgcaccgatcgcccttcccaacagttgcgcagcctgaatggcgaatgggacgcgccctgtagcggcgcattaagcgcggcgggtgtggtggttacgcgcagcgtgaccgctacacttg
ccagcgccctagcgcccgctcctttcgctttcttcccttcctttctcgccacgttcgccggctttccccgtcaagctctaaatcgggggctccctttagggttccgatttagtgctttacggcacctcgaccccaaaaaacttgatt
agggtgatggttcacgtagtgggccatcgccctgatagacggtttttcgccctttgacgttggagtccacgttctttaatagtggactcttgttccaaactggaacaacactcaaccctatctcggtctattcttttgatttataa
gggattttgccgatttcggcctattggttaaaaaatgagctgatttaacaaaaatttaacgcgaattttaacaaaatattaacgcttacaatttaggtggcacttttcggggaaatgtgcgcggaacccctatttgtttatttttc
taaatacattcaaatatgtatccgctcatgagacaataaccctgataaatgcttcaataatattgaaaaaggaagagtatgagtattcaacatttccgtgtcgcccttattcccttttttgcggcattttgccttcctgtttttgct
cacccagaaacgctggtgaaagtaaaagatgctgaagatcagttgggtgcacgagtgggttacatcgaactggatctcaacagcggtaagatccttgagagttttcgccccgaagaacgttttccaatgatgagcacttttt
aaagttctgctatgtggcgcggtattatcccgtattgacgccgggcaagagcaactcggtcgccgcatacactattctcagaatgacttggttgagtactcaccagtcacagaaaagcatcttacggatggcatgacagtaa
gagaattatgcagtgctgccataaccatgagtgataacactgcggccaacttacttctgacaacgatcggaggaccgaaggagctaaccgcttttttgcacaacatgggggatcatgtaactcgccttgatcgttgggaacc
ggagctgaatgaagccataccaaacgacgagcgtgacaccacgatgcctgtagcaatggcaacaacgttgcgcaaactattaactggcgaactacttactctagcttcccggcaacaattaatagactggatggaggcgg
ataaagttgcaggaccacttctgcgctcggcccttccggctggctggtttattgctgataaatctggagccggtgagcgtgggtctcgcggtatcattgcagcactggggccagatggtaagccctcccgtatcgtagttatct
acacgacggggagtcaggcaactatggatgaacgaaatagacagatcgctgagataggtgcctcactgattaagcattggtaactgtcagaccaagtttactcatatatactttagattgatttaaaacttcatttttaattt
aaaaggatctaggtgaagatcctttttgataatctcatgaccaaaatcccttaacgtgagttttcgttccactgagcgtcagaccccgtagaaaagatcaaaggatcttcttgagatcctttttttctgcgcgtaatctgctgctt
gcaaacaaaaaaaccaccgctaccagcggtggtttgtttgccggatcaagagctaccaactctttttccgaaggtaactggcttcagcagagcgcagataccaaatactgtccttctagtgtagccgtagttaggccaccac
ttcaagaactctgtagcaccgcctacatacctcgctctgctaatcctgttaccagtggctgctgccagtggcgataagtcgtgtcttaccgggttggactcaagacgatagttaccggataaggcgcagcggtcgggctgaac
ggggggttcgtgcacacagcccagcttggagcgaacgacctacaccgaactgagatacctacagcgtgagctatgagaaagcgccacgcttcccgaagggagaaaggcggacaggtatccggtaagcggcagggtcgg
aacaggagagcgcacgagggagcttccagggggaaacgcctggtatctttatagtcctgtcgggtttcgccacctctgacttgagcgtcgatttttgtgatgctcgtcaggggggcggagcctatggaaaacgccagcaac
gcggcctttttacggttcctggccttttgctggccttttgctcacatgttctttcctgcgttatcccctgattctgtggataaccgtattaccgcctttgagtgagctgataccgctcgccgcagccgaacgaccgagcgcagcga
gtcagtgagcgaggaagcgattgcacgttgaagcgttacctgttaggtaacgtagttgagctgtgcaatctatttttaacaacgatagttgagctgtcacagaatgtgacgttgaAGTCGTTGTTGGACTCATTCAA
CCATGGGCTCCAGGGGCGGAAGAGCAGGCAAGCTGCGGACACAAATTTCTCGACAGTAGGGATATGTTTCACTACGTGTGGCGTCCTGGACAGGGTTCCTAGCAGTC
GTATGGGTTCGCTCTTACAGGACACCTGGTATTCGCAATGGTCTCACGAGGCCCTGCTCATGGCTCTTAGTCAACTCTACTCATGCAATACCGTATTCCGAGGCGCTCCG
ATAAATGGCTATGTGTGCTTGTCGACATCAGTCGGCAAAGAGACAAATATAGTGCTAGCTACGCAACAAGTATTAACGAACGGCCATTCGAATAGTGGAAGACATCGC
CTACTCCGTGCCAAAGCCTCACGAAAAGCCACGTATAGGGTTGCTGATGGTGCACACCAGGGCTGTTCTATCCAGCTCCATATGTGGAGAAGGCAACGTAGTCAGTTAC
GGAGTTATCCCACCTTAGTATTACTCGATCTGATGTCTGCACGCTGAGGTCTCCCACAGTCGTCGTGTCAACAATAGAGAGTCAGGCTCTTGCTATTCTATCCAAGGGCA
CAGTCCAACTACCCAGTCACCAGAGGGACCTGAAGGTGTTCTCAGTGCCGAGATCCTATCGGTTACTGTTACCAGGCGAGAGCAAGCTGTCGGGGATTTGAGGTGCAC
TCCGGATACCCACAAAACGTGATCGCAGTCCATCCCTGAGACATTTTAGCGTAACTGGGCTGAGTATTCTACTGAGCTGGCGTAGCGTTGAACCCTTCAGGACGATAGA
TTCGGGTAAAGACGGTCATCGGGGGCTTATACAGCGGACTACAACTCTAGAGCTTGAATGACATACTCATCTCTATACATTCTCGAACAGCCTATCAGCGAGTCGATTAC
AGAGGTCCCT**ACGTTGA**agcgttacctgttaggtaacgtagttgagctgtagggacgatttttgctcacattagttgagctgtcacagaatgtgacgttgaagatgtgagcccctttcgccag

References

1. Praetorius F*, et al.* (2017) Biotechnological mass production of DNA origami. *Nature* 552:84-87.
2. Pinto C, Kasaciunaite K, Seidel R, & Cejka P (2016) Human DNA2 possesses a cryptic DNA unwinding activity that functionally integrates with BLM or WRN helicases. *Elife* 5:e18574.
3. Chan YW & West SC (2018) GEN1 endonuclease: Purification and nuclease assays. *Methods in Enzymology*, eds Spies M & Malkova A (Academic Press, Burlington, New York), Mechanisms of DNA Recombination and Genome Rearrangements, pp 527-542.
4. Young SJ*, et al.* (2020) MutSβ stimulates Holliday junction resolution by the SMX complex. *Cell Rep.* 33:108289.
5. Wyatt HDM, Sarbajna S, Matos J, & West SC (2013) Coordinated actions of SLX1-SLX4 and MUS81-EME1 for Holliday junction resolution in human cells. *Mol. Cell* 52:234-247.
6. Wyatt HDM, Laister RC, Martin SR, Arrowsmith CH, & West SC (2017) The SMX DNA repair tri-nuclease. *Mol. Cell* 65:848-860.
7. Kingston IJ, Yung JS, & Singleton MR (2011) Biophysical characterization of the centromere-specific nucleosome from budding yeast. *J. Biol. Chem.* 286:4021-4026.
8. Kurat CF, Yeeles JTP, Patel H, Early A, & Diffley JFX (2017) Chromatin controls DNA replication origin selection, lagging-strand synthesis, and replication fork rates. *Molec. Cell* 65:117-130.
